# Development and Characterization of Agar–Starch-Based Bioplastic Films

**DOI:** 10.3390/polym18111321

**Published:** 2026-05-27

**Authors:** Alaa Alnatsheh, Birce Dikici, Rishikesh Srinivasaraghavan Govindarajan, Daewon Kim

**Affiliations:** 1 Department of Mechanical Engineering, Embry-Riddle Aeronautical University (ERAU), Daytona Beach, FL 32114, USA; alnatsha@my.erau.edu; 2 Department of Mechanical Engineering, Florida Polytechnic University, Lakeland, FL 33805, USA; rsrinivas@floridapoly.edu; 3 Department of Aerospace Engineering, Embry-Riddle Aeronautical University (ERAU), Daytona Beach, FL 32114, USA; kimd3c@erau.edu

**Keywords:** agar–starch bioplastics, biopolymer composites, mechanical properties, thermal analysis, FTIR characterization, water absorption, soil biodegradation, bio-based packaging

## Abstract

This study investigates agar–starch composite bioplastic films formulated with five agar-to-starch ratios (1:1, 2:1, 3:1, 4:1, and 5:1) to evaluate how composition influences material performance. Films were produced by solution casting with glycerol as a plasticizer and characterized through tensile testing (ASTM D882-18), DSC, TGA, FTIR, water absorption measurements, physical property assessment, and biodegradability tests including water, UV, and soil degradation. Mechanical results showed that the 3:1 formulation (A3S1) exhibited the highest tensile strength (2.78 MPa) with moderate elongation (57.25%), while the 1:1 formulation (A1S1) showed the greatest flexibility (76.38% elongation) but lower strength (2.07 MPa). Thermal analysis indicated improved thermal stability with increasing agar content, with onset degradation temperatures ranging from 42.89 °C to 51.84 °C and melting points from 99 °C to 108 °C. FTIR spectra showed no new major absorption bands, with only minor shifts in selected bands, indicating component interactions without evidence of new chemical bond formation. Films with higher starch content displayed increased thickness, weight per area, and water absorption. Overall, adjusting agar–starch ratios produced distinct combinations of mechanical, thermal, and physical properties, with the 3:1 ratio offering the best balance of strength and water resistance. All formulations showed measurable biodegradation under water, UV, and soil conditions, indicating environmental degradability.

## 1. Introduction

The growing environmental concerns surrounding petroleum-based plastics have intensified research into biodegradable alternatives derived from renewable resources [1]. Although significant progress has been made in developing bioplastics from natural polymers, achieving an optimal balance of mechanical performance, thermal stability, and cost-effectiveness continues to pose challenges for large-scale adoption [1].

Agar and starch are two widely investigated biopolymers due to their availability and biodegradability. Agar-based films, derived from red seaweed, offer strong gel-forming ability and better water resistance than many other natural polymers [2]. However, agar films tend to be brittle, sensitive to moisture, and relatively expensive due to processing costs. In contrast, starch-based bioplastics are low-cost, abundant, and highly biodegradable [3,4,5], but they typically suffer from poor mechanical properties and high water absorption, limiting their commercial viability [6,7].

Efforts to optimize agar-based materials further highlight these trade-offs. Hernández et al. [8] developed agar–glycerol films with varying agar (0.5–8%) and glycerol (0.31–10.7%) concentrations using the solution casting method, reporting that improvements in tensile strength were often accompanied by reduced flexibility. Their findings underscore the need to balance rigidity and ductility, as higher plasticizer content increases elongation but decreases tensile strength.

Beyond agar-based systems, extensive research has also focused on starch as a low-cost bioplastic material. Abdullah et al. [5] demonstrated that starch sources such as corn, cassava, and potato yield films with different mechanical and thermal properties, highlighting the variability and limitations of starch-based systems. Although starch offers excellent biodegradability and availability, pure starch films typically require reinforcement to improve their strength and water resistance.

To address these limitations, reinforcement strategies have been widely examined. Tan et al. [9] investigated chitosan-reinforced starch films and showed that plasticizers such as glycerol can enhance flexibility, though careful optimization is needed to maintain mechanical integrity. Similarly, composite approaches have gained traction across multiple biopolymer systems. Mostafavi et al. [6] reviewed various agar-based composites—including agar–starch, agar–chitosan, and agar–protein blends—and concluded that composite formulations consistently outperform single-polymer films by enabling targeted tuning of mechanical and thermal properties.

Earlier composite-focused studies support these trends. For example, Akter et al. [10] reported that adding chitosan to starch-based films improved thermal stability and char formation, demonstrating the composition-dependent nature of biopolymer blends. Collectively, these findings indicate that blending complementary biopolymers is an effective strategy for overcoming the inherent weaknesses of individual components.

Water absorption behavior has also been widely examined in composite bioplastic development. Taharuddin et al. [11] reported that incorporating agar into thermoplastic starch composites increased moisture uptake due to the hydrophilic nature of the components, demonstrating the challenge of maintaining mechanical integrity in high-moisture environments.

Research has also focused on polymer interactions within composite systems. Amariei et al. [12] developed agar–alginate membranes plasticized with glycerol and showed through FTIR and mechanical analysis that physical blending creates composite structures primarily through molecular entanglement and hydrogen bonding, rather than chemical reactions. This aligns with the framework established by Kuo [13], who demonstrated that intermolecular hydrogen bonding and chain entanglement can significantly improve blend compatibility without requiring chemical modifications, supporting the feasibility of simple, scalable processing routes.

Agar–starch blend films have been previously reported [6,14,15]. Wu et al. [14] examined agar–potato starch films with agar contents ranging from 0% to 30% of the total polymer content, using pure agar as a control, and characterized them using FTIR, XRD, SEM, moisture absorption, tensile properties, and water vapor permeability. Mostafavi et al. [6] also reported that starches from different sources have been blended with agar, with film structure depending on the starch-to-agar ratio. In this context, the present study investigates five fixed agar:corn starch ratios, ranging from 1:1 to 5:1, within an agar-rich composition range.

This study does not introduce a new modification strategy; rather, it presents an integrated, ratio-based evaluation of five agar:corn starch formulations (1:1, 2:1, 3:1, 4:1, and 5:1), all prepared using a consistent method and evaluated within a unified testing framework. The films were characterized in terms of physical, mechanical, thermal, and FTIR properties, as well as water absorption and environmental degradation behavior. Environmental degradation was assessed through UV exposure, water immersion, and soil burial. The aim of this study is to determine how formulation ratio influences multiple property domains within the same agar–corn starch system and to identify the formulation that provides the most balanced overall performance.

## 2. Materials and Methods

### 2.1. Materials

Agar (LB Agar, Molecular Biology Grade) was obtained from Research Products International, RPI (Mt Prospect, IL, USA). Glycerol (Hi-LR™, GRM081) was purchased from HiMedia Laboratories Pvt. Ltd. (Maharashtra, India). Corn starch (food grade) was obtained from Whole Foods Market. Distilled water was used in all experiments.

### 2.2. Sample Preparation

Film preparation followed the solution-casting method described by Alnatsheh & Dikici [16], with modifications to enable systematic testing of agar–starch ratios. The compositions of the agar–starch composite films are summarized in Table 1. For all formulations, the total solids content was kept constant at 8 g, glycerol at 2.5 g, water at 160 g, and total batch mass at 170.5 g.

Water, agar, and starch were first mixed manually for 3 min to aid initial dispersion and dissolution, during which the temperature was raised gradually to approximately 75 °C. The mixture was then stirred using a magnetic stirrer at 500 rpm for 5 min, while the temperature was increased to approximately 95 °C, until a homogeneous film-forming solution was obtained. The optimal casting point was reached when the solution became translucent, slightly thickened, and free of visible undissolved particles.

The film-forming solutions were manually cast into identical silicone molds (28 cm × 25 cm) and dried at 23 ± 2 °C for 48 h to obtain the final films. For each formulation, the entire batch was cast into a single mold, ensuring consistent casting volume and surface area across all samples. The viscosity of the film-forming solutions was not measured in this study.

### 2.3. Physical Property Measurements

The prepared films were evaluated for thickness, weight-to-area ratio, and bulk density. Films were cut into 2 × 2 cm squares using a scalpel to ensure clean edges. Weight was measured at room temperature (23 ± 2 °C) using a digital analytical balance (TORBAL AGZN220, Scientific Industries, Inc., Bohemia, NY, USA; precision ±0.0001 g, capacity 220 g). Thickness was measured at four locations for each formulation using a digital micrometer (precision 0.001 mm). Thickness and weight-to-area ratio data were analyzed using one way ANOVA, followed by Tukey’s HSD post hoc test at *p* < 0.05. The bulk density of agar and starch powders was determined using a 50 mL pycnometer (TQC B.V. VF 2098, Capelle aan den Ijssel, The Netherlands; ISO 2811/DIN 53 217/ASTM D1475) [17,18,19]. The pycnometer was filled with powder and gently tapped five times to minimize voids. Bulk density ($\rho_{b}$) was calculated as:

(1) $$\rho_{b}=\frac{m_{1}-m_{2}}{V}$$

where $m_{1}$ is the mass of the filled pycnometer (g), $m_{2}$ is the mass of the empty pycnometer (g), and V is the pycnometer volume (50 mL). Five measurements were taken for each material. All analyses were conducted under laboratory conditions (23 ± 2 °C, 55% relative humidity).

### 2.4. Mechanical Property Testing

Tensile strength, elongation at break, and Young’s modulus of the films were measured according to ASTM D882-18 [20] using a universal testing machine (AMETEK, CS225, Largo, FL, USA). For each formulation, five rectangular specimens (20 × 100 mm) with a gauge length of 60 mm were tested under laboratory conditions (23 ± 2 °C, 55% relative humidity) at a crosshead speed of 5 mm/min. For mechanical calculations, the measured thickness of each specimen was used to determine its cross-sectional area; thus, tensile strength was calculated using specimen-specific dimensions rather than average film thickness values. Results are reported as mean ± standard deviation. Statistical differences among formulations in tensile strength, elongation at break, and Young’s modulus were evaluated using one-way ANOVA, followed by Tukey’s HSD post hoc test at *p* < 0.05.

### 2.5. Thermal Analysis Methods

#### 2.5.1. DSC Analysis

Differential scanning calorimetry (DSC) analysis was performed using a Mettler Toledo DSC3 analyzer (Mettler-Toledo, LLC, Columbus, OH, USA) to investigate the thermal transitions of the films. Samples (5–10 mg) were sealed in aluminum pans and heated from 0 to 150 °C under a nitrogen atmosphere at a rate of 10 °C/min, in accordance with ASTM E793/E794 [21,22]. The onset temperature (T_o_) and peak melting temperature (T_p_) were determined from the thermograms. Three replicates were analyzed for each formulation to ensure reproducibility. Thermal transition data were analyzed using one-way ANOVA, followed by Tukey’s HSD post hoc test at *p* < 0.05.

#### 2.5.2. TGA

Thermal behavior of the films was evaluated using a Mettler Toledo TGA2 instrument (Mettler-Toledo, LLC, Columbus, OH, USA). Samples (15–20 mg) were heated from 30 to 800 °C at a rate of 5 °C/min under a nitrogen atmosphere (flow rate: 20 mL/min). These conditions are consistent with common protocols for biopolymer analysis, where heating rates of 5–20 °C/min under nitrogen are typically used [7,23,24]. Weight loss as a function of temperature was recorded to determine the thermal degradation profile and assess the thermal stability of the films.

### 2.6. Water Absorption Testing

Water absorption of the films was measured following methods reported for starch-based bioplastics [9,25,26]. Samples (2 × 2 cm) were weighed to determine their initial dry mass ($W_{initial}$) and then immersed in distilled water for 90 min. At 10 min intervals (10, 20, 30, 40, 50, and 60 min) and at 90 min, specimens were removed, gently blotted to remove surface water, weighed ($W_{wet}$), and returned to the water.

Water absorption (%) was calculated as:

(2) $$\mathrm{Water}\mathrm{absorption}\%=\;\frac{W_{wet}-W_{initial}}{W_{initial}}\;\times 100$$

Three specimens were tested per formulation, and average values were reported. For statistical comparison among formulations, the 90 min water absorption values were analyzed using one-way ANOVA, followed by Tukey’s HSD post hoc test at *p* < 0.05.

The test was conducted in distilled water to provide a consistent baseline for comparing water uptake across formulations.

### 2.7. Fourier-Transform Infrared (FTIR) Analysis

FTIR spectroscopy was used to identify functional groups and assess chemical interactions in the films. Spectra were recorded using an Agilent Cary 630 FTIR spectrometer (Agilent Technologies Inc. Headquarters, Santa Clara, CA, USA) with a spectral resolution of ≤2 cm^−1^ over a range of 4000–500 cm^−1^. Measurements were performed on both the composite films and the individual raw materials (agar, starch, and glycerol) used in the formulations.

### 2.8. Environmental Degradation Testing

Environmental degradation of the agar–starch films was evaluated using three controlled laboratory exposure tests. The films were subjected to UV radiation, water immersion, and soil burial, representing common environmental conditions that influence the stability and performance of bioplastic materials.

#### 2.8.1. UV Radiation Testing

**Figure 1:**
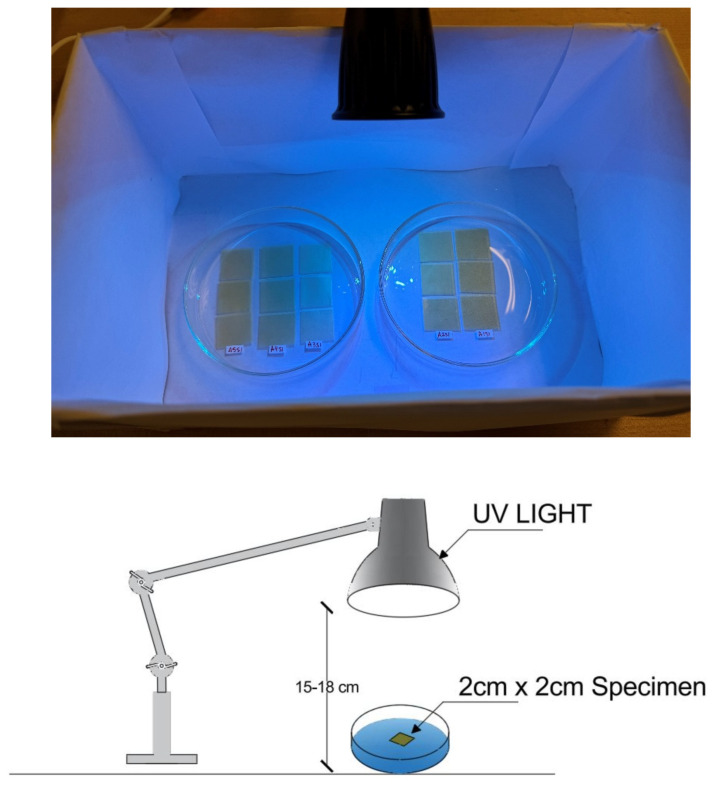
UV irradiation testing setup with specimen under UV light.

Film samples (2 cm × 2 cm) were exposed to UVA and UVB light (λ = 280–400 nm) using a Reptilamp^®^ UV lamp (ZhongShan Maineng Lighting LTD (ZSMNL), Xiaolan, Zhongshan, China) for 48 h, with samples positioned 15–18 cm from the light source. To minimize external light interference, the test setup was enclosed within a box; the lamp and sample arrangement are shown in Figure 1.

Color measurements were performed before and after UV exposure using a colorimeter (FRU Global, Shenzen, Guangdong, China) to quantify color changes in the CIELAB color space. The CIELAB system provides a standardized method for objective color evaluation and is widely used for monitoring material color alterations [27], following definitions outlined in ISO/CIE 11664-4:2019 [28]. In this system, L* represents lightness (0 = black, 100 = white), a* represents the red–green axis (positive = red, negative = green), and b* represents the yellow–blue axis (positive = yellow, negative = blue).

For each formulation, three samples were measured, and average L*, a*, and b* values were calculated. The total color difference (ΔE*) was determined using:

(3) $$\Delta E^{*}=\sqrt{(\Delta L^{*}{)}^{2}+(\Delta a^{*}{)}^{2}+(\Delta b^{*}{)}^{2}}$$

where ΔL*, Δa*, and Δb* represent the differences between the initial and final L*, a*, and b* values, respectively.

#### 2.8.2. Water Degradation Testing

Film samples were weighed, and color parameters (L*, a*, and b*) were measured using a colorimeter prior to immersion. Samples were then immersed in distilled water at room temperature (23 ± 2 °C) and 55% relative humidity for 7 days.

After immersion, the samples were removed, gently blotted with tissue paper to eliminate surface water, and air-dried at room temperature for an additional 7 days. Final mass and color measurements were subsequently performed to evaluate mass changes and color variations. The total color difference (ΔE*) was calculated as described previously.

The percentage mass loss was determined using the following equation:

(4) $$\mathrm{Mass}\mathrm{Loss}\%=\;\frac{W_{i}-W_{f}}{W_{i}}\;\times 100$$

where $W_{i}$ is the initial dry mass of the film before immersion and $W_{f}$ is the final dry mass after water immersion and drying to constant weight.

Although the films may undergo temporary swelling during immersion, mass measurements were performed after drying to isolate irreversible material loss associated with water-induced degradation.

#### 2.8.3. Soil Biodegradation Testing

The third environmental exposure test involved soil burial to assess biodegradation under natural decomposition conditions. Commercial potting soil was used as the testing medium and was supplemented with an organic fertilizer to enhance microbial activity and accelerate biodegradation processes.

Film samples (2 cm × 2 cm) were individually weighed and buried at a depth of 5 cm in the prepared soil within plastic containers. The containers were maintained under controlled laboratory conditions at a temperature of 23 ± 2 °C and 55% relative humidity for a period of 4 months. Soil moisture was monitored and maintained throughout the test duration.

At the end of the exposure period, the soil was carefully excavated to retrieve all remaining sample fragments. Retrieved samples were photographed and visually categorized according to their physical degradation state: intact (original shape maintained), perforated (visible holes with overall structure preserved), fragmented (broken into multiple pieces), or not recovered (no material detected).

All recoverable fragments were gently cleaned, air-dried to constant weight, and weighed to determine the total recoverable mass for subsequent mass loss calculations.

## 3. Results and Discussion

### 3.1. Physical Property Results

**Figure 2:**
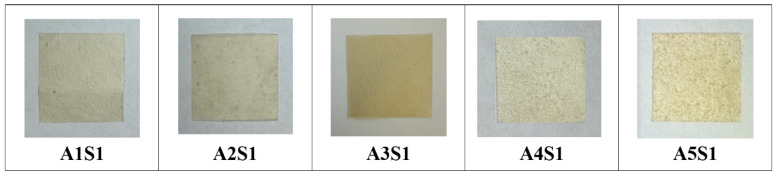
Photographs of agar–starch film samples (2 cm × 2cm).

Figure 2 presents the prepared agar–starch composite films prior to physical property characterization.

#### 3.1.1. Bulk Density Analysis of Powders

Pycnometer-based bulk density measurements indicated that agar powder had a higher bulk density (0.7177 ± 0.0041 g/cm^3^) compared to starch powder (0.6651 ± 0.0114 g/cm^3^). The smaller standard deviation for agar powder reflects more consistent packing behavior, while the larger variability observed for starch powder suggests greater particle heterogeneity.

#### 3.1.2. Thickness Analysis

**Figure 3:**
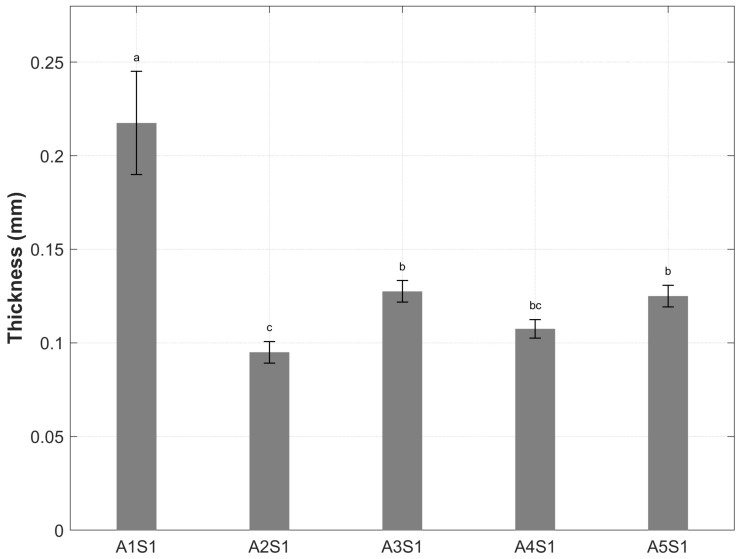
Thickness of agar–starch composite films. Bars represent mean ± standard deviation. Different letters indicate significant differences (*p* < 0.05).

The thickness of the prepared agar–starch films is presented in Figure 3. Film thickness varied across formulations, ranging from 0.095 to 0.2175 mm, with no clear linear relationship to the agar-to-starch ratio. One-way ANOVA showed a significant effect of formulation on film thickness (*p* < 0.05). Based on Tukey’s HSD test, A1S1 showed the highest thickness and differed significantly from all other formulations. A3S1 and A5S1 were not significantly different from each other, and A2S1 did not differ significantly from A4S1.

All formulations were prepared with the same total batch composition and cast under consistent processing conditions. As summarized in Table 1, water (160 g), glycerol (2.5 g), total solids (8 g), and total batch mass (170.5 g) were kept constant across all formulations. The film-forming solutions were cast into the same mold; therefore, differences in thickness were not attributable to variations in formulation mass or casting area.

Previous studies have shown that thickness in solution-cast polymer films can be influenced by solution behavior during casting, in addition to formulation composition. Hidayati et al. [29] reported no direct relationship between chitosan:polyvinyl alcohol ratio and film thickness. Hazrol et al. [30] observed thickness changes in starch-based films with formulation variations, and Kramar et al. [31] reported that solution flow and film spreading during casting influenced final film thickness.

Accordingly, the thickness differences observed here are interpreted more cautiously as reflecting variations in film formation and spreading behavior during casting and drying, rather than a direct effect of agar–starch ratio alone. The viscosity of the film-forming solutions was not measured in this study; therefore, viscosity-related effects cannot be directly assessed. To account for thickness variation in mechanical testing, the measured thickness of each individual specimen was used to calculate cross-sectional area, rather than applying a single average thickness value.

#### 3.1.3. Weight per Area Analysis

**Figure 4:**
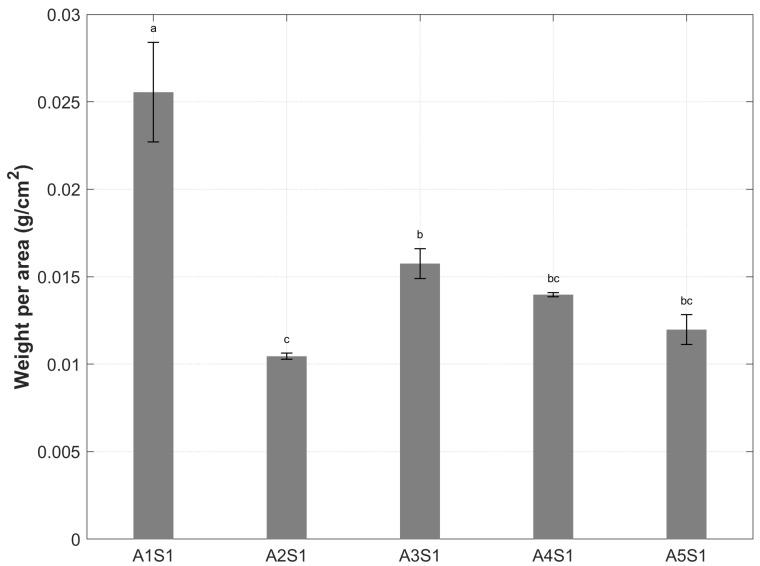
Weight per area of agar–starch composite films. Bars represent mean ± standard deviation. Different letters indicate significant differences (*p* < 0.05).

The weight per area of the agar–starch films is presented in Figure 4. Overall, weight per area followed a trend similar to film thickness across most formulations, with the exception of A5S1. Although differences were observed among formulations, no clear linear relationship with agar–starch ratio was identified. One-way ANOVA indicated a significant effect of formulation on weight per area (*p* < 0.05). Based on Tukey’s HSD test, A1S1 exhibited the highest weight per area and differed significantly from all other formulations. A3S1 was significantly higher than A2S1 but did not differ significantly from A4S1 or A5S1, while A2S1 did not differ significantly from A4S1 or A5S1.

Because all formulations were prepared with the same total batch composition and cast into the same mold under consistent conditions, the observed differences in weight per area are attributed to variations in film formation among formulations rather than to composition alone. Weight per area is presented here as a comparative physical property in relation to the measured thickness values; no specific mechanism is proposed.

### 3.2. Mechanical Property Results

#### 3.2.1. Tensile Strength Analysis

**Figure 5:**
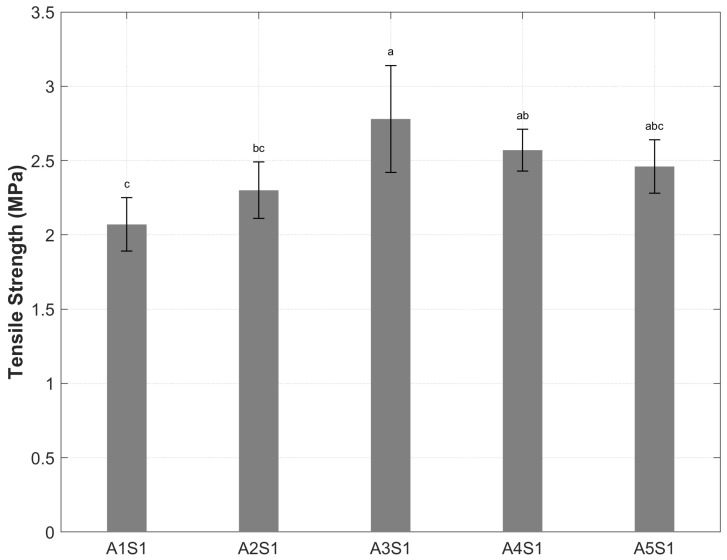
Ultimate tensile strength of agar–starch films with varying weight ratios. Bars represent mean ± standard deviation. Different letters indicate significant differences (*p* < 0.05).

Tensile strength values for the agar–starch films are presented in Figure 5. The tensile strength ranged from 2.07 to 2.78 MPa across the five formulations, with A3S1 (3:1 agar-to-starch ratio) exhibiting the highest value (2.78 ± 0.36 MPa). One-way ANOVA indicated a significant effect of formulation on tensile strength (*p* < 0.05). Based on Tukey’s HSD test, A3S1 was significantly higher than A2S1 and A1S1, and A4S1 was significantly higher than A1S1, while the remaining pairwise differences were not significant.

Tensile strength increased from A1S1 to A3S1, then decreased at A4S1 and A5S1, indicating that tensile performance did not increase continuously with agar content within the studied composition range.

Wu et al. [14] reported that agar addition can increase tensile strength in starch-based films through intermolecular interactions and the formation of a denser network structure, which is consistent with the trend observed in the present study.

#### 3.2.2. Elongation at Break Analysis

**Figure 6:**
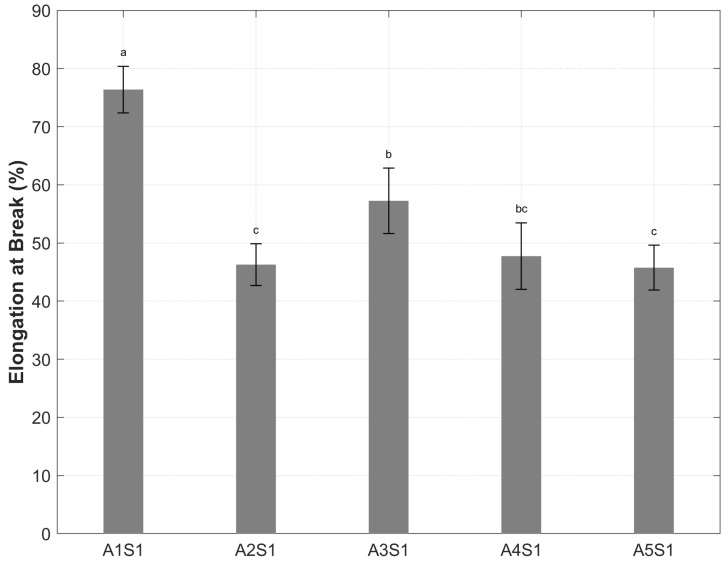
Elongation at break of agar–starch films with varying weight ratios. Bars represent mean ± standard deviation. Different letters indicate significant differences (*p* < 0.05).

Elongation at break values for the agar–starch films are presented in Figure 6, ranging from 45.75% to 76.38%. A1S1 (1:1 agar-to-starch ratio) exhibited the highest elongation (76.38 ± 4.01%). One-way ANOVA indicated a significant effect of formulation on elongation at break (*p* < 0.05). Based on Tukey’s HSD test, A1S1 was significantly higher than all other formulations, while A3S1 was significantly higher than A2S1 and A5S1; the remaining differences were not significant.

Elongation decreased from A1S1 to A2S1, increased at A3S1, and then decreased again at A4S1 and A5S1, indicating a non-linear relationship with agar content. The high elongation observed for A1S1 corresponds to its lower tensile strength, reflecting a trade-off between flexibility and strength across the formulations.

Wu et al. [14] reported that elongation in agar–starch films reflects the balance between polymer chain mobility and network restriction. In the present study, elongation generally decreased with increasing agar content, suggesting reduced chain mobility in more agar-rich formulations.

#### 3.2.3. Young’s Modulus Analysis

**Figure 7:**
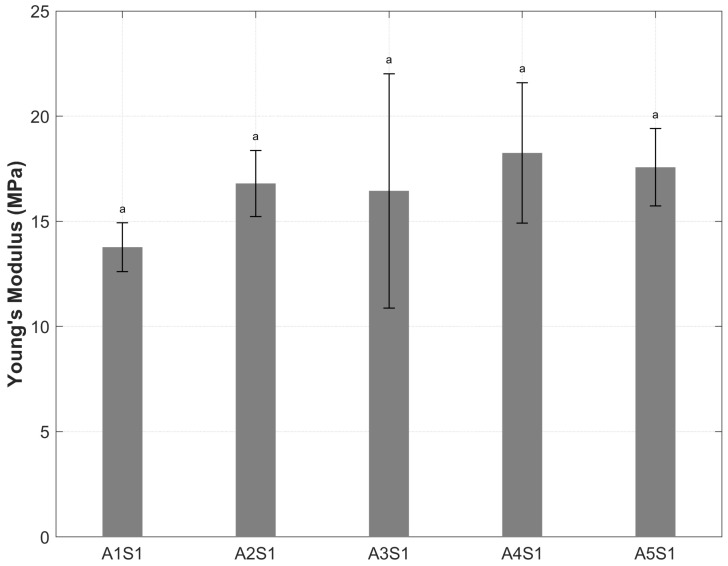
Young’s modulus of agar–starch films with varying weight ratios. Bars represent mean ± standard deviation. Different letters indicate significant differences (*p* < 0.05).

Young’s modulus values for the agar–starch films are presented in Figure 7, ranging from 13.77 to 18.25 MPa. A4S1 exhibited the highest mean modulus (18.25 ± 3.34 MPa), while A1S1 showed the lowest (13.77 ± 1.16 MPa). One-way ANOVA indicated no significant effect of formulation on Young’s modulus (*p* > 0.05), suggesting that stiffness remained relatively consistent across the tested compositions.

**Figure 8:**
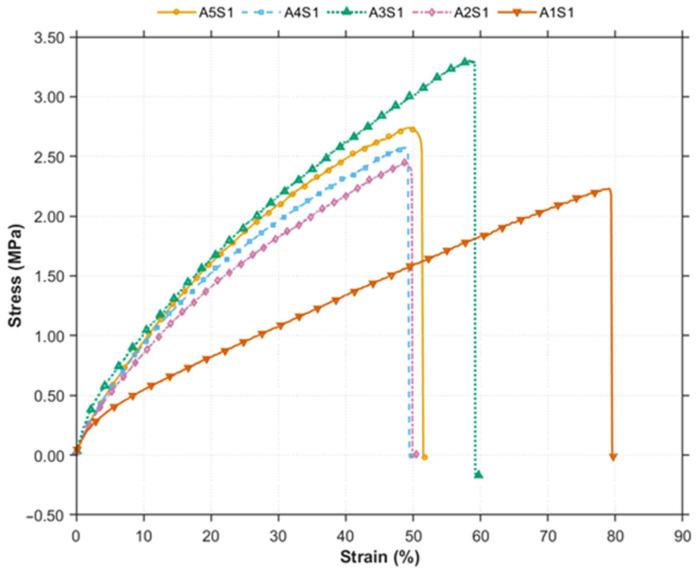
Comparison of representative stress–strain curves for film formulations A5S1–A1S1.

The lower modulus of A1S1 is consistent with its higher elongation at break, indicating reduced stiffness and greater flexibility. Representative stress–strain curves for all formulations are shown in Figure 8. Table 2 summarizes the mechanical properties of agar–starch films.

Compared to reported values for pure starch and agar films [7,8,9,23], the agar–starch composites in this study exhibit intermediate mechanical behavior, balancing stiffness and flexibility.

### 3.3. Thermal Analysis Results

#### 3.3.1. Differential Scanning Calorimetry (DSC) Analysis

DSC thermograms of the agar–starch films exhibited endothermic transitions for all formulations. The onset temperature ($T_{o}$) and peak melting temperature ($T_{p}$) values are summarized in Table 3.

The onset temperature decreased with increasing starch content, from 51.84 °C for A5S1 (5:1 agar-to-starch ratio) to 42.89 °C for A1S1 (1:1), indicating that higher starch content lowered the temperature at which thermal transitions began. One-way ANOVA indicated a significant effect of formulation on onset temperature (*p* < 0.05). Tukey’s HSD test showed that A5S1 was significantly higher than A3S1, A2S1, and A1S1, while A4S1 did not differ significantly from either A5S1 or the lower-ratio formulations.

**Figure 9:**
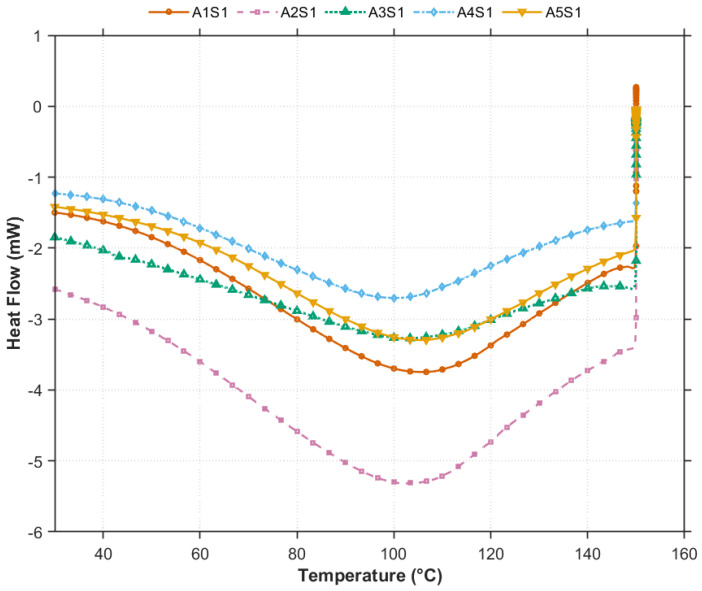
DSC thermograms of agar–starch films with varying agar-to-starch ratios.

The highest melting temperature was observed for A5S1 (107.99 °C), followed by A4S1 (103.32 °C), whereas A1S1, A2S1, and A3S1 showed similar values (~99 °C). However, ANOVA indicated no significant effect of formulation on melting temperature (*p* > 0.05). Representative DSC thermograms are shown in Figure 9.

These results are consistent with Nagar et al. [32], who reported a non-linear dependence of melting temperature on agar content in starch–agar films. They attributed lower melting temperatures at low agar content to disruption of starch crystallinity, whereas higher agar content promoted more ordered structures requiring greater thermal energy. In the present study, formulation had a clearer effect on onset temperature than on peak melting temperature.

#### 3.3.2. Thermogravimetric Analysis (TGA)

**Figure 10:**
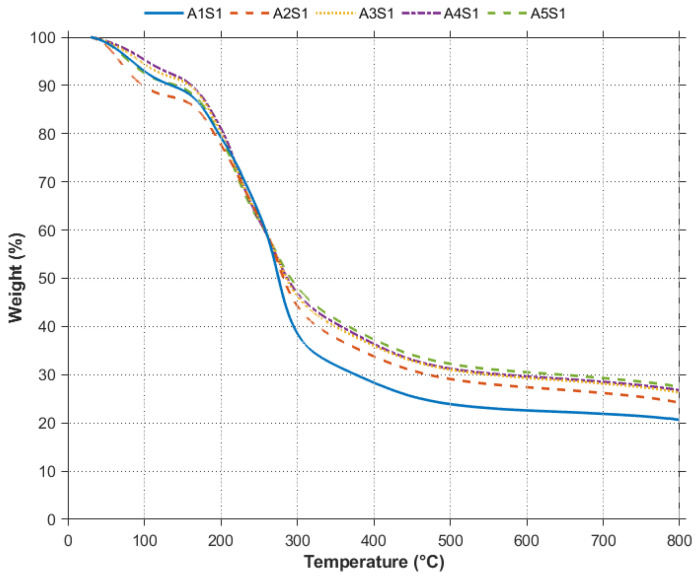
TGA curves of agar–starch films with varying ratios.

**Figure 11:**
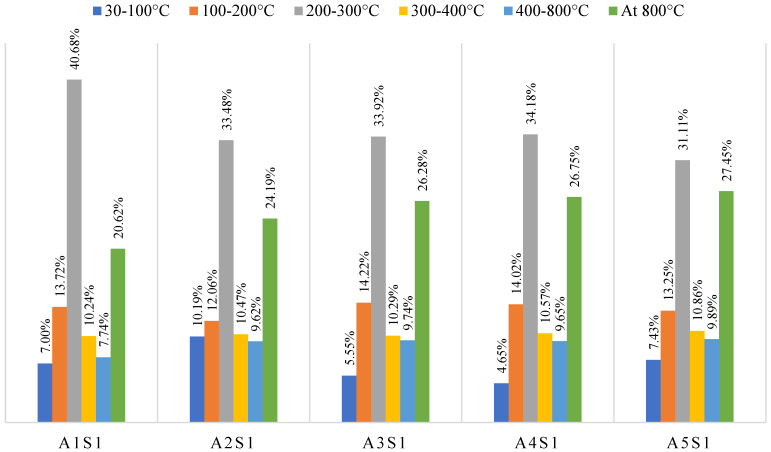
Thermal degradation behavior of agar–starch formulations based on weight loss percentages.

The thermal degradation behavior of the agar–starch bioplastic films exhibited multistage decomposition patterns dependent on composition, as shown in Figure 10. All formulations, with agar-to-starch ratios ranging from 1:1 to 5:1, displayed characteristic multi-step degradation profiles. The corresponding transition temperatures are summarized in Table 4, and the weight-loss patterns are presented in Figure 11.

The degradation process can be described in distinct temperature regions:

- 30–100 °C: An initial weight loss of 4.65–10.19% was attributed to the evaporation of free and bound water within the bioplastic matrix. Films with higher starch content exhibited greater moisture loss than agar-rich formulations, reflecting the higher water absorption capacity of starch.
- 100–200 °C: Weight loss of 12.06–14.22% corresponds to continued evaporation of bound moisture and volatile components. Minimal variation among formulations suggests this stage is dominated by moisture removal rather than polymer degradation.
- 200–300 °C: This region represents the primary thermal decomposition stage, with weight loss ranging from 31.11% to 40.68%. The A1S1 formulation exhibited the highest weight loss (40.68%), while A5S1 showed the lowest (31.11%), indicating that starch contributes more significantly to thermal degradation in this temperature range.
- 300–400 °C: Additional weight loss of 10.24–10.86% occurred due to decomposition of more thermally stable components. Agar-rich formulations exhibited slightly higher weight loss (A5S1: 10.86%) than starch-rich films (A1S1: 10.24%), suggesting that agar decomposes at higher temperatures and demonstrates greater thermal stability.
- 400–800 °C: Gradual weight loss of 7.74–9.89% corresponds to decomposition of carbonized residues. At these temperatures, compositional differences become less significant because both polymers have largely decomposed. The final char residue at 800 °C increased with agar content, ranging from 20.62% for A1S1 to 27.45% for A5S1, indicating that agar promotes the formation of more thermally stable carbonaceous structures during degradation.

### 3.4. Water Absorption Test Results

**Figure 12:**
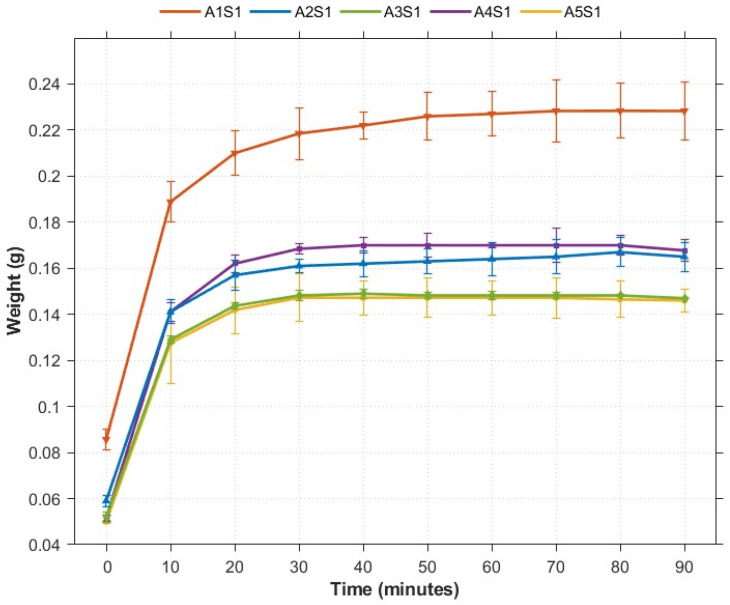
Weight of agar–starch formulations at different immersion times.

All formulations exhibited rapid initial water uptake within the first 20–30 min, followed by a slower phase approaching equilibrium. Weight measurements at different immersion times are shown in Figure 12.

At 90 min, water absorption values were 171.68% (A1S1, b), 221.84% (A2S1, a), 148.56% (A3S1, b), 225.78% (A4S1, a), and 185.78% (A5S1, ab). Thus, A2S1 and A4S1 showed significantly higher uptake, A1S1 and A3S1 lower uptake, and A5S1 intermediate behavior. All formulations approached equilibrium within approximately 50–60 min.

The rapid initial uptake followed by gradual equilibration is characteristic of polysaccharide-based films. Similar behavior has been reported for starch-based systems, where water absorption occurs predominantly within the first 20–40 min before stabilizing [30,33]. The higher absorption values observed in the present study (148–225%) further reflect the strong hydrophilicity of the agar–starch matrix.

Water absorption did not vary linearly with agar:starch ratio. Notably, A3S1 exhibited lower uptake than the neighboring A2S1 and A4S1 formulations, suggesting that water uptake was influenced by formulation-dependent film structure rather than composition alone. This behavior is consistent with the mechanical results, where A3S1 also showed the highest tensile strength. However, as no diffusion modeling or molecular-level analysis was performed, this interpretation is limited to a comparative assessment under the present test conditions.

### 3.5. FTIR Analysis

**Figure 13:**
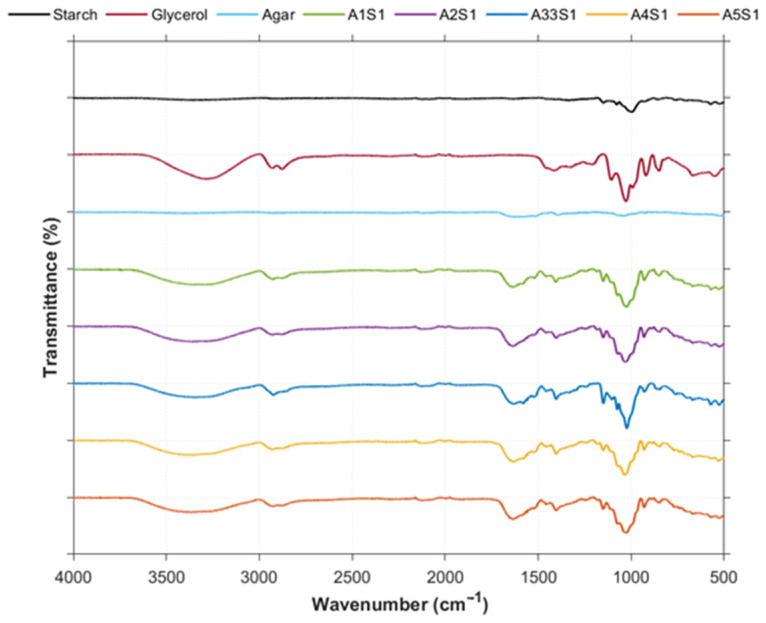
Stacked FTIR spectra of agar–starch films and raw materials.

FTIR spectra were recorded over the range of 4000–500 cm^−1^ for all agar–starch formulations, along with pure corn starch, agar, and glycerol. The stacked spectra are shown in Figure 13, and the main peak positions are summarized in Table 5.

The pure components exhibited characteristic absorption bands in the O–H stretching, C–H stretching, and fingerprint regions. Corn starch showed a broad O–H band at 3268 cm^−1^, C–H stretching at 2923 cm^−1^, and main fingerprint bands at 1148 and 995.6 cm^−1^. Glycerol exhibited O–H stretching at 3272 cm^−1^, C–H stretching at 2930 cm^−1^, and a C–O band at 1028 cm^−1^, while agar showed a broad O–H band at 3158 cm^−1^, C–H stretching at 2925 cm^−1^, and a C–O band at 1041 cm^−1^.

No new major absorption bands were observed in the blended films, indicating that film formation occurred through physical blending rather than new chemical bond formation [12,23,34]. In the blended films, the O–H stretching band appeared in the range 3271–3278 cm^−1^ and the C–H stretching band at 2926–2931 cm^−1^. A band in the 1624–1636 cm^−1^ region, commonly attributed to absorbed water in starch-based systems, was present in all formulations [7,14].

The fingerprint region showed bands at 1241–1244, 1149–1150, 1031–1023, 930, and 846–847 cm^−1^, corresponding to C–O and C–O–C vibrations typical of starch–agar systems [7,12,14,23]. Small shifts in band position were observed between the raw materials and blended films, particularly in the O–H and fingerprint regions. Particularly, A3S1 exhibited the lowest band position in the 1031–1023 cm^−1^ region (1023 cm^−1^), compared to 1025–1031 cm^−1^ for the other formulations. These shifts indicate intermolecular interactions among components within a physically blended system.

### 3.6. Environmental Degradation Analysis

Environmental degradation tests revealed distinct changes in film properties across all agar–starch formulations under different exposure conditions. Each degradation pathway exhibited composition-dependent responses.

#### 3.6.1. UV Exposure Results

After 48 h of UV exposure, all formulations exhibited measurable color changes, indicating photo-oxidative degradation. The wavelength range used (280–315 nm) corresponds to UV-B radiation, which is known to induce material degradation under natural conditions [35]. Colorimetric analysis revealed varying degrees of color alteration across the different agar–starch ratios. As summarized in Table 6, the total color difference (ΔE*) ranged from 2.33 to 6.31, based on the average of three measurements per formulation.

The A2S1 formulation exhibited the largest color change (ΔE* = 6.31), while A5S1 showed the smallest alteration (ΔE* = 2.33). Most samples exhibited a decrease in lightness, indicated by negative ΔL* values, except A4S1, which maintained its lightness. Positive Δa* values across all formulations indicate a shift toward red coloration, while positive Δb* values suggest yellowing. All formulations showed ΔE* values greater than 2.0, indicating perceptible color changes across the entire range of agar–starch compositions tested. The intermediate ratios (A2S1, A1S1, and A3S1) demonstrated greater color sensitivity compared to formulations with higher agar content (A4S1 and A5S1).

UV radiation induces polymer degradation primarily through photo-oxidative mechanisms, leading to polymer chain scission, chromophore formation, and consequent color changes [35]. Photon absorption can cause photodissociation, breaking polymer chains into smaller fragments. Bertolini et al. [36] reported that UV irradiation of cassava and corn starch resulted in the formation of carbonyl and carboxyl groups along polymer chains, accompanied by yellowing and molecular weight reduction. These processes align with established degradation pathways involving depolymerization, crosslinking, and the formation of unsaturated bonds within the polymer matrix.

Similar UV-induced degradation behavior has been reported in other biodegradable polymer systems. Sun et al. [37] investigated PBAT/PLA films subjected to prolonged UV exposure and observed surface chalking and mass loss that intensified with exposure time, indicating polymer breakdown. The observable color changes in the agar–starch films after 48 h of UV exposure suggest that these materials undergo comparable photo-oxidative degradation mechanisms.

#### 3.6.2. Water Immersion Test Results

**Figure 14:**
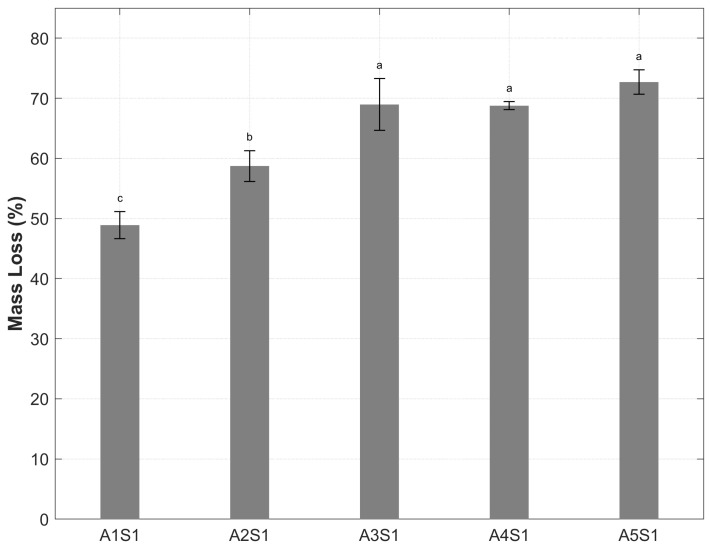
Mass loss percentages of agar–starch formulations after water immersion test. Bars represent mean ± standard deviation. Different letters indicate significant differences (*p* < 0.05).

After 7 days of water immersion followed by air drying, mass loss measurements revealed formulation-dependent degradation behavior (Figure 14). Mass loss ranged from 48.9 ± 2.3% to 72.7 ± 2.0% across all formulations, indicating substantial water-induced degradation.

One-way ANOVA indicated a significant effect of formulation on mass loss (*p* < 0.05). Tukey’s HSD test showed that A3S1, A4S1, and A5S1 formed the higher mass-loss group, A2S1 was intermediate, and A1S1 had significantly lower mass loss than all other formulations.

Similar behavior has been reported for starch-based bioplastics, where initial water uptake is followed by material loss during prolonged immersion [38]. In the present study, the measured mass loss after drying reflects degradation occurring during water exposure.

Water immersion also resulted in measurable color changes (Table 7). A3S1 showed the smallest change (ΔE* = 6.35), while A1S1, A2S1, A4S1, and A5S1 exhibited higher ΔE* values (17.92–23.66). Lightness (L*) generally increased, while a* and b* values decreased, indicating reduced red and yellow tones. These changes suggest structural and compositional alterations during degradation, including possible leaching of colored components.

All films showed visible shrinkage, increased transparency, and greater brittleness after the immersion–drying cycle. This behavior is consistent with the known water sensitivity of starch-based systems [39] and aligns with the substantial mass loss observed.

#### 3.6.3. Soil Burial Test Results

After four months of soil burial, the agar–starch films exhibited substantial physical degradation. Three specimens per formulation were buried, but not all were recovered at the end of the test. The results are summarized in Table 8. Of the 15 specimens, 11 were recovered with measurable final mass, while 4 (26.7%) were not recovered. A3S1 and A4S1 each yielded three recovered specimens, A2S1 and A5S1 each yielded two, and A1S1 yielded one.

Among the recovered specimens, average weight loss was 73.0% for A1S1 (n = 1), 55.0 ± 17.0% for A2S1 (n = 2), 63.8 ± 1.7% for A3S1 (n = 3), 61.1 ± 6.3% for A4S1 (n = 3), and 62.6 ± 19.3% for A5S1 (n = 2). Due to the unequal number of recovered specimens and the limited sample size for some formulations, these data are interpreted descriptively and were not subjected to statistical comparison.

**Figure 15:**
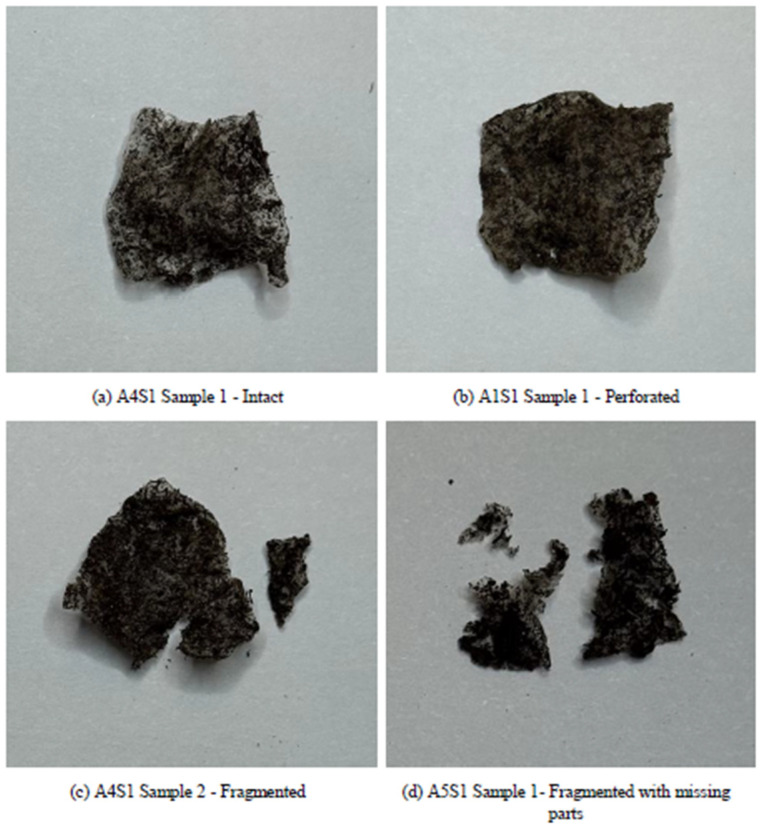
Examples of fragmented samples retrieved from soil burial sites, demonstrating advanced stages of biodegradation with adhered soil particles.

Recovered samples frequently exhibited adhered soil particles that could not be fully removed without damaging the degraded films, introducing uncertainty in final mass measurements. Based on visual observations, specimens were classified as intact, perforated, fragmented, or unrecovered (Figure 15). Unrecovered specimens were not considered definitive evidence of complete biodegradation but rather indicative of advanced degradation and/or fragmentation beyond recovery.

Similar degradation behavior has been reported for starch-based and related bioplastics under soil burial conditions [38,40]. These studies support the interpretation that non-recovery after prolonged burial may be associated with extensive degradation, although it does not confirm complete biodegradation under the present conditions.

### 3.7. Integrated Formulation and Property Relationship

The results demonstrate that formulation ratio governs the balance among mechanical performance, thermal behavior, and water sensitivity in agar–starch films. FTIR analysis showed no new absorption bands, with only minor shifts in selected regions, indicating composition-dependent intermolecular interactions without new chemical bond formation. Notably, A3S1 exhibited the lowest band position in the 1031–1023 cm^−1^ region, suggesting differences in interaction patterns among formulations.

Mechanically, A3S1 exhibited the highest tensile strength (2.78 ± 0.36 MPa), while A1S1 showed the greatest elongation at break (76.38 ± 4.01%), confirming a trade-off between strength and flexibility. Thermally, A5S1 showed the highest onset temperature, whereas melting temperature did not differ significantly among formulations. In water immersion, A3S1 exhibited the lowest absorption (148.07%), while A4S1 showed the highest (225.19%), indicating that water sensitivity did not vary linearly with composition.

The tensile strength values (2.07–2.78 MPa) fall below those of conventional plastics such as LDPE (≈9.93 MPa) and PET (55–79 MPa) [41,42], but are comparable to thermoplastic starch (~2.6 MPa) [43]. The higher elongation values observed (45.75–76.38%) indicate greater flexibility than PLA, which typically shows limited elongation [42]. These results place the films within the performance range of polysaccharide-based materials suitable for low-load applications where biodegradability is prioritized.

No single formulation maximized all properties. Among those tested, A3S1 provided the most balanced performance, combining relatively high tensile strength with low water uptake and moderate thermal stability. This assessment is based on comparative trends and does not represent a molecular-level interaction analysis.

## 4. Conclusions

This study evaluated agar–starch composite films across five formulation ratios to assess their physical, mechanical, thermal, water absorption, and biodegradation behavior. The results showed that increasing agar content improved thermal stability, while higher starch content enhanced flexibility.

FTIR analysis indicated physical blending with composition-dependent intermolecular interactions and no evidence of new chemical bond formation. All formulations exhibited measurable degradation under water, UV, and soil burial conditions, confirming their environmental degradability.

Among the tested formulations, A3S1 provided the most balanced combination of properties, while A5S1 showed the highest thermal stability and A1S1 the greatest flexibility. These findings highlight the importance of formulation ratio in tailoring performance within agar–starch systems.

Future work should focus on detailed structural analysis and the use of reinforcement or modification strategies to enhance mechanical performance for targeted applications.

## Figures and Tables

**Table 1:** Composition of agar–starch composite films.

Sample	Agar: Starch Ratio	Agar (g)	Starch (g)	Total Solids (g)	Agar (*w*/*w*%)	Starch (*w*/*w*%)	Glycerol (g)	Water (g)	Total (g)
A1S1	1:1	4.00	4.00	8	2.35%	2.35%	2.5	160	170.5
A2S1	2:1	5.33	2.67	8	3.13%	1.57%	2.5	160	170.5
A3S1	3:1	6.00	2.00	8	3.52%	1.17%	2.5	160	170.5
A4S1	4:1	6.40	1.60	8	3.75%	0.94%	2.5	160	170.5
A5S1	5:1	6.67	1.33	8	3.91%	0.78%	2.5	160	170.5

**Table 2:** Mechanical Properties of Agar–Starch Films.

Sample	Tensile Strength (MPa)	Elongation at Break (%)	Young’s Modulus (MPa)
A1S1	2.07 ± 0.18 ^c^	76.38 ± 4.01 ^a^	13.77 ± 1.16 ^a^
A2S1	2.30 ± 0.19 ^bc^	46.28 ± 3.58 ^c^	16.80 ± 1.57 ^a^
A3S1	2.78 ± 0.36 ^a^	57.25 ± 5.60 ^b^	16.45± 5.57 ^a^
A4S1	2.57 ± 0.14 ^ab^	47.74 ± 5.73 ^bc^	18.25 ± 3.34 ^a^
A5S1	2.46 ± 0.18 ^abc^	45.75 ± 3.85 ^c^	17.57 ± 1.84 ^a^

Values are reported as mean ± standard deviation. Different letters indicate significant differences (*p* < 0.05).

**Table 3:** Thermal properties of agar–starch composite films determined by DSC.

Sample ID	Agar:Starch Ratio	Onset Temp (°C)	Melting Temp (°C)
A1S1	1:1	42.89 ± 1.48 ^b^	99.75 ± 7.12 ^a^
A2S1	2:1	43.77 ± 2.17 ^b^	99.17 ± 2.07 ^a^
A3S1	3:1	44.10 ± 2.71 ^b^	98.90 ± 5.16 ^a^
A4S1	4:1	45.44 ± 1.05 ^ab^	103.32 ± 2.06 ^a^
A5S1	5:1	51.84 ± 3.29 ^a^	107.99 ± 2.33 ^a^

Values are reported as mean ± standard deviation. Different letters indicate significant differences (*p* < 0.05).

**Table 4:** Summary of TGA data for agar–starch composite films.

Degradation Step	Temperature Range	A1S1	A2S1	A3S1	A4S1	A5S1
Initial moisture loss	30–100 °C	7.00%	10.19%	5.55%	4.65%	7.43%
Onset of polymer degradation	100–200 °C	13.72%	12.06%	14.22%	14.02%	13.25%
Primary degradation phase	200–300 °C	40.68%	33.48%	33.92%	34.18%	31.11%
Secondary degradation phase	300–400 °C	10.24%	10.47%	10.29%	10.57%	10.86%
Final carbonization	400–800 °C	7.74%	9.62%	9.74%	9.65%	9.89%
Residual char at 800 °C	-	20.62%	24.19%	26.28%	26.75%	27.45%

**Table 5:** Main FTIR peak positions of raw materials and agar–starch films.

Sample	O–H Stretching (cm^−1^)	C–H Stretching (cm^−1^)	H–O–H Bending of Water (cm^−1^)	C–O Band (cm^−1^)
Agar	3158	2925	—	1041
Glycerol	3272	2930	—	1028
Starch	3268	2923	1636	1076
A1S1	3271	2928	1635	1025
A2S1	3273	2931	1636	1030
A3S1	3276	2926	1624	1023
A4S1	3278	2930	1636	1031
A5S1	3275	2930	1636	1026

**Table 6:** CIELAB Color Changes After 48 h UV Exposure.

Sample	Initial Properties				Final Properties				Difference			
	L*	a*	b*	Color	L*	a*	b*	Color	ΔL*	Δa*	Δb*	ΔE*
A1S1	83.84	1.85	28.71		80.25	3.67	31.61		−3.59	1.82	2.90	4.96
A2S1	86.29	0.62	23.20		82.58	2.48	27.95		−3.71	1.86	4.75	6.31
A3S1	89.41	−0.62	14.00		87.34	0.34	18.35		−2.07	0.96	4.35	4.91
A4S1	84.28	1.42	27.30		84.30	1.78	24.81		0.02	0.36	−2.49	2.52
A5S1	82.57	2.59	31.30		81.63	3.27	29.28		−0.94	0.68	−2.02	2.33

The colored cells illustrate the actual visual color of the film samples before and after 48 h UV exposure, corresponding to the reported CIELAB (L*, a*, b*) values.

**Table 7:** CIELAB color change in agar–starch formulations after water immersion.

Sample	Initial Color	Final Color	ΔL*	Δa*	Δb*	ΔE*
A1S1	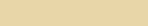	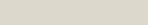	1	−1.55	−17.8	17.92
A2S1	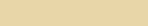	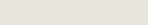	5.41	−1.38	−18.83	19.65
A3S1	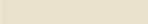	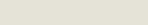	0.31	−0.13	−6.33	6.35
A4S1	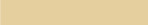	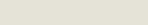	5.97	−1.86	−21.42	22.37
A5S1	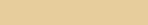	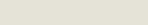	6.33	−2.07	−22.69	23.66

The colored cells represent the visual appearance of the film samples before and after water immersion, corresponding to the measured CIELAB (L*, a*, b*) values.

**Table 8:** Soil burial results of agar–starch films after 4 months.

Formulation	Recoverable Specimens, n	Weight Loss (%)	Mean ± SD (%)	Retrieval Outcome
A1S1	1	73.0	—	Two specimens not recovered
A2S1	2	67.0, 43.0	55.0 ± 17.0	One specimen not recovered
A3S1	3	62.8, 65.8, 62.8	63.8 ± 1.7	All specimens recovered
A4S1	3	56.4, 58.6, 68.3	61.1 ± 6.3	All specimens recovered
A5S1	2	76.2, 48.9	62.6 ± 19.3	One fragmented specimen recovered; one specimen not recovered

## Data Availability

The original contributions presented in this study are included in the article. Further inquiries can be directed to the corresponding author.

## Abbreviations

The following abbreviations are used in this manuscript:

ANOVA	Analysis of variance
ASTM	American Society for Testing and Materials
DSC	Differential Scanning Calorimetry
FTIR	Fourier-Transform Infrared
MPa	Megapascal
UV	Ultraviolet
UVA	Ultraviolet A
UVB	Ultraviolet B
TGA	Thermogravimetric Analysis
wt.%	Weight Percent
*w*/*w*%	Weight by Weight Percent
*v*/*v*	Volume by Volume
